# Monitoring of Caffeine Consumption Effect on Skin Blood Properties by Diffuse Reflectance Spectroscopy

**DOI:** 10.33549/physiolres.930000.74.161

**Published:** 2025-02-01

**Authors:** Matija MILANIC, Rok HREN, Jost STERGAR, Urban SIMONCIC

**Affiliations:** 1 Faculty of Mathematics and Physics, University of Ljubljana, Ljubljana, Republic of Slovenia; 2 Jožef Stefan Institute, Ljubljana, Republic of Slovenia; 3 Institute of Mathematics, Physics, and Mechanics, Ljubljana, Republic of Slovenia; 4 Syreon Research Institute, Budapest, Hungary



Physiol Res 2024 Mar 11;73(1):47–56. doi: 10.33549/physiolres.935138.

PMID: 38466004

